# A protocol for a systematic review and meta-analysis of strategies to quantify or eliminate catastrophic costs due to tuberculosis

**DOI:** 10.12688/wellcomeopenres.17521.1

**Published:** 2022-03-15

**Authors:** Paula P. Carballo-Jimenez, Sumona Datta, Rubén Aguirre-Ipenza, Matthew J. Saunders, Luz Quevedo Cruz, Carlton A. Evans

**Affiliations:** 1 IFHAD: Innovation For Health And Development, Department of Infectious Disease, Imperial College London, London, UK; 2 IPSYD: Innovación Por la Salud Y Desarrollo, Asociación Benéfica Prisma, Lima, Peru; 3 IFHAD: Innovation For Health And Development, Laboratory of Research and Development, Universidad Peruana Cayetano Heredia, Lima, Peru; 4 Department of Clinical Sciences, Liverpool School of Tropical Medicine, Liverpool, UK; 5 Universidad Continental, Lima, Peru

**Keywords:** systematic review, meta-analysis, catastrophic costs, tuberculosis

## Abstract

**Background:** The World Health Organization strategy to “End TB” by 2030 includes the milestone of no affected households facing catastrophic costs due to tuberculosis (TB). Costs due to TB are usually defined as catastrophic if they exceed 20% of the pre-disease annual household income. Several countries have conducted national TB cost surveys but strategies to quantify and eliminate catastrophic costs are incompletely defined.

**Methods: **Publications related to strategies to quantify and eliminate catastrophic costs will be identified by searching three electronic databases (PubMed - Medline, Scopus and Web of Science) together with reference lists from pertinent publications. We will screen eligible studies, extract data, and assess the risk of bias with the quality assessment tool from the National Heart, Lung, and Blood Institute. Discrepancies will be resolved by discussion between the reviewers. If we find sufficient comparable studies quantifying strategies to eliminate catastrophic costs then a meta-analysis will be performed. This systematic review and meta-analysis is registered with the PROSPERO database (CRD42022292410).

**Conclusion:** This systematic review and meta-analysis aims to rigorously assess the evidence for strategies to quantify or eliminate catastrophic costs due to TB.

## Introduction

Since records began, tuberculosis (TB) has killed more people than any other infectious disease globally. TB is strongly associated with poverty because TB principally affects poorer people in poorer regions ^1^. Furthermore, costs due to TB disease, diagnosis and treatment can all worsen poverty ^1^.

Costs due to TB are usually assessed at the level of the household and include direct out-of-pocket expenditures and also the indirect costs of lost income due to TB, including before TB was diagnosed or treated. These costs due to TB have been quantified using diverse strategies ^2^ including:

- prospective recording of costs versus retrospective recall;
- retrospective recall of costs at the start of treatment and repeatedly at various intervals during treatment versus assessing costs over the past month at one randomly selected time during treatment;
- paper versus electronic data collection;
- locally-developed versus standardised data collection instruments; and
- diverse strategies to assess pre-disease household income that are used as the denominator for assessing whether costs due to TB were catastrophic.

In 2011 the World Health Organisation (WHO) together with the Japan Anti-Tuberculosis Association (JATA) developed a tool to estimate costs due to TB. This venture led to the creation of a standardised handbook for conducting TB patient cost surveys that has been used in several countries ^2^.

As costs due to TB increase, the risk of adverse TB treatment outcomes (principally treatment non-completion) increases. Indeed, we found that in Peruvian shantytowns when costs due to TB exceeded 20% of the pre-illness income of that household, then the risk of adverse treatment outcomes (treatment non-completion, treatment failure or death during treatment) were more likely than favourable treatment outcome (cure of successful treatment outcome) ^3^. Similar findings have been reported in Brazil and Moldova ^4, 5^. Consequently, costs due to TB are usually considered to be catastrophic if they exceed a threshold of 20% of the pre-illness household annual income ^6^, although other thresholds have been used occasionally ^7, 8^.

The WHO End TB Strategy has three principal milestones, including aiming to ensure that there are zero households facing catastrophic costs due to TB by 2030 ^9^. This milestone is generally believed to require sufficient political action that TB-affected patients and their TB-affected households can:

- reduce direct costs of out-of-pocket expenditures due to TB;
- reduce indirect costs by maintaining their income as much as possible despite TB;

and also where necessary

- receive socioeconomic support to reduce the impact of costs due to TB.

Preventing catastrophic costs due to TB has been prioritised in global policy in order to mitigate the impoverishing effects of TB ^1^ and also in order to increase the likelihood that patients with TB will be able to afford to complete TB care sufficiently to be permanently cured and return to good health ^10^.

Despite the consensus that catastrophic costs due to TB should be prevented, there is remarkably little clarity concerning how this may best be achieved. For example, from first principles it seems logical that interventions including the following may reduce costs due to TB, towards eliminating catastrophic costs due to TB.

- Improved health systems and active case finding searching for people with TB disease (instead of passive case finding, waiting for them to present to and be diagnosed by health facilities) may more often diagnose TB earlier in the disease, whilst it is less severe and has caused less costs.
- Education, public health promotion, stigma reduction, laws and other measures may further reduce the indirect costs of lost employment due to TB.
- Information, improved health systems and universal health coverage may help to reduce the direct out-of-pocket expenditures caused by TB disease.
- Providing home-based care versus community-based clinic care versus hospital-based care in order to potentially reduce direct and indirect costs due to TB.
- TB-specific socioeconomic support for people with TB disease may mitigate and/or reimburse their direct and indirect costs due to TB.
- Existing socioeconomic support systems (such as microcredit or cash transfer interventions to reduce extreme poverty) may be TB-sensitive, or be made more sensitive to the needs of people living with TB, for example by adding TB disease to their eligibility criteria.
- Socioeconomic development may decrease poverty sufficiently to reduce the risk that costs due to TB reach the threshold for catastrophic costs.
- Reductions in poverty, under-nutrition, HIV, and other factors together possibly with improved public health systems may reduce the incidence of TB and hence indirectly reduce the incidence of catastrophic costs due to TB.

We have modelled the potential global effects of TB-specific versus TB-sensitive interventions ^11^ and have in Peru been prospectively evaluating the health and economic effects of TB-specific socioeconomic interventions for TB-affected households ^12– 16^. Related studies have been reported in other settings ^17^, and ecological analyses ^18^ and modelling ^19^ studies have assessed the impact of social protection interventions on TB. For the current research, in order to inform public health policy, we aim to complete a systematic review and meta-analysis of these and other approaches to quantify or eliminate catastrophic costs due to TB.

## Objectives

The objectives of this systematic review and meta-analysis are to rigorously assess the evidence for strategies to quantify or eliminate catastrophic costs due to TB

## Review question

What evidence informs strategies to quantify or eliminate catastrophic costs due to TB?

## Methods

This systematic review and meta-analysis will follow the Preferred Reporting Items for Systematic Reviews and Meta-Analyses (PRISMA-P) checklist. The protocol is registered in the PROSPERO database 2022 CRD42022292410.

### Eligibility criteria

*Inclusion criteria.* Studies concerning the quantification or elimination of catastrophic household costs due to TB, including any type of TB (pulmonary or extrapulmonary; drug-susceptible or drug-resistant; whether or not complicated by comorbidities such as associated HIV-infection).

*Exclusion criteria.* Studies that could not inform strategies to achieve the WHO target of eliminating catastrophic costs due to TB because they only quantified:

- out-of-pocket expenditure costs without considering indirect costs of lost income; or
- monetary costs without assessing these costs as a proportion of household income; or
- catastrophic costs at a population level without considering the proportion of individual households that experienced catastrophic costs.

### Population

The population to be included in this systematic review and meta-analysis is TB-affected households i.e. patients with TB and the people living with them.

### Intervention/Exposure

Interventions will include any strategies aiming to mitigate or eliminate catastrophic costs due to TB e.g. TB active case finding (versus standard of care passive case finding); socio-economic support (compared with standard of care without socio-economic support); or home-based care (compared with standard of care in hospital).

### Comparison

The comparison / control condition will be standard of care (without any intervention).

### Outcome

The proportion of households with catastrophic costs due to TB.

### Information sources

Three electronic databases will be searched: PubMed, Scopus and Web of Science. We will also search reference lists from relevant publications.

### Search strategy

We will use the following search terms:

Pubmed: ((tuberculosis[MeSH Terms]) OR (tuberculosis OR koch disease* OR TB[Title/Abstract]))

AND (catastrophic cost* OR catastrophic household cost*[Title/Abstract])

Scopus: TITLE-ABS-KEY ( ( tuberculosis OR "koch disease" OR tb ) AND ( catastrophic AND cost* ) )

Web of science: (tuberculosis OR Koch disease* OR TB) AND (catastrophic AND cost* ) (All Fields)

### Measures of effect

The proportion of households with catastrophic costs due to TB will be the main measure. For continuous or categorical data outcomes, mean or rate differences between the catastrophic cost intervention group and the control group will be used. For dichotomous data outcomes, odds ratio, relative risk, and/or absolute risk will be used. For data measured on the same scale and the same unit, weighted mean differences will be used, otherwise standardised mean differences will be used. The 95% confidence intervals of these measures will also be assessed.

### Data extraction

Studies will be selected for inclusion from the electronic databases selected using the search strategy. We will also review the references cited by these publications to find other relevant articles. Two reviewers will independently review potentially relevant publication titles, then abstracts and finally full-text publications for eligibility. Discrepancies will be resolved by discussion and when necessary independent consideration by another reviewer. The following data will be extracted from each publication:

- catastrophic cost results (e.g. the proportion of households with catastrophic costs due to TB);
- study characteristics;
- methodological characteristics;
- variables known to be related to catastrophic costs (e.g. drug-resistant TB, household income).

The data will be extracted in CSV format that will be uploaded to the Rayyan software to screen duplicate documents as well in a Microsoft Excel spreadsheet document. The study selection process will be documented using the PRISMA flow diagram. Heterogeneity of data will be assessed if there is enough suitable data to perform a meta-analysis. A shared cloud-based spreadsheet will log all edits and who makes them.

### Type of studies

We will include all types of studies that inform the review objectives, without any restriction. For example: observational quantitative, qualitative, and mixed methods studies; intervention studies including randomised controlled trials; reviews; editorials; perspectives; and mathematical modelling studies will be extracted.

### Risk of bias (quality) assessment

A quality assessment tool from the National Heart, Lung, and Blood Institute (NHLBI) will be used to generate an overall rating for the quality of each study of “good”, “fair”, or “poor” (see https://www.nhlbi.nih.gov/health-topics/studyquality-assessment-tools). Depending on pilot work after the data have been extracted, an alternative tool may be used such as Version 2 of the Cochrane risk-of-bias tool for randomised trials. We anticipate that the quality assessment tool for case control studies may be most appropriate. These plans may be modified if necessary, as adaptations to the progress of the systematic review.

### Strategy for data synthesis

Catastrophic cost results will be presented as percentages, costs as means or medians, and as statistically significant (P<0.05) or not. Additionally, odds ratios (or relative risks) comparing study groups will be calculated for interventions potentially affecting catastrophic costs.

### Meta-analysis

If we find suitable intervention studies, then we will assess the heterogeneity of the data with I ^2^ statistics and a Forest plot graph. All data will be analysed using Stata Software version 16.0 (Stata Corporation LLC, College Station, Texas, USA). The meta-analyses will include pooled odds ratios of comparable studies calculating the respective weighted means of these ratios, including weighted confidence intervals.

## Ethics and dissemination

Approval from an Ethics committee is not required for this systematic review and meta-analysis that includes analysis of only anonymous unlinked data. We intend to present this work at conferences and to publish it in an international peer-reviewed open-access journal.

## Discussion

The COVID-19 pandemic is markedly increasing TB disease, adverse TB outcomes, catastrophic costs due to TB and poverty ^20^. We hope that this systematic review will help to strengthen the evidence base for quantifying catastrophic costs due to TB. We also hope that this systematic review and meta-analysis will help to inform strategies for reducing or potentially eliminating catastrophic costs due to TB, towards ending TB.

## Data availability

### Underlying data

No data are associated with this article.

### Reporting guidelines

Harvard Dataverse: PRISMA-P checklist for “A protocol for a systematic review and meta-analysis of strategies to quantify or eliminate catastrophic costs due to tuberculosis”, https://doi.org/10.7910/DVN/JS3GVY/DKK8LN ^21^.

Data are available under the terms of the Creative Commons Zero "No rights reserved" data waiver (CC0 1.0 Public domain dedication).
