# Interventions aiming to eliminate catastrophic costs due to tuberculosis: a protocol for a systematic review and meta-analysis

**DOI:** 10.12688/wellcomeopenres.17521.2

**Published:** 2022-11-03

**Authors:** Paula P. Carballo-Jimenez, Sumona Datta, Rubén Aguirre-Ipenza, Matthew J. Saunders, Luz Quevedo Cruz, Carlton A. Evans

**Affiliations:** 1 IFHAD: Innovation For Health And Development, Department of Infectious Disease, Imperial College London, London, UK; 2 IPSYD: Innovación Por la Salud Y Desarrollo, Asociación Benéfica Prisma, Lima, Peru; 3 IFHAD: Innovation For Health And Development, Laboratory of Research and Development, Universidad Peruana Cayetano Heredia, Lima, Peru; 4 Department of Clinical Sciences, Liverpool School of Tropical Medicine, Liverpool, UK; 5 Universidad Continental, Lima, Peru

**Keywords:** systematic review, meta-analysis, catastrophic costs, tuberculosis

## Abstract

***Background*** **:** People with tuberculosis disease and their household members may suffer direct out-of-pocket expenses and indirect costs of lost income. These tuberculosis-related costs can worsen poverty, make tuberculosis treatment completion unaffordable, impair quality of life and increase the risk of death. Costs due to tuberculosis are usually defined as catastrophic if they exceed 20% of the pre-disease annual household income. The World Health Organisation strategy to “End TB” and the United Nations Sustainable Development Goals include the target that no households should face catastrophic costs due to tuberculosis. However, there is limited evidence and policy concerning how this global priority of eliminating catastrophic costs due to tuberculosis should be achieved. This systematic review and meta-analysis aims to address this knowledge gap.

***Methods*** **: **Publications assessing interventions that aimed to eliminate catastrophic costs will be identified by searching three electronic databases (PubMed, Scopus and Web of Science) together with reference lists from pertinent publications. We will screen eligible studies, extract data, and assess the risk of bias with the quality assessment tool from the National Heart, Lung, and Blood Institute. Discrepancies will be resolved by discussion between the reviewers. If we find sufficient comparable studies quantifying strategies to eliminate catastrophic costs then a meta-analysis will be performed. This systematic review and meta-analysis is registered with the PROSPERO database (CRD42022292410).

***Conclusion*** **:** This systematic review and meta-analysis aims to rigorously assess the evidence for strategies to eliminate catastrophic costs due to tuberculosis.

## Introduction

Since records began, tuberculosis (TB) has killed more people than any other infectious disease globally. TB is strongly associated with poverty because TB principally affects poorer people in poorer regions and TB disease, diagnosis and treatment related costs can all worsen poverty ^1^.

Costs due to TB are usually assessed at the level of the household and include direct out-of-pocket expenditures and also the indirect costs of lost income due to TB, including before TB was diagnosed or treated. These costs due to TB have been quantified using diverse strategies including ^2^:

- prospective recording of costs versus retrospective recall over brief periods or retrospective recall over prolonged periods;
- characterisation of actual costs throughout the TB illness versus assessing costs over one short period (usually one month) at one randomly selected time during treatment and then extrapolating these costs to the duration of the entire illness;
- paper versus electronic data collection;
- locally developed cost data collection tools versus internationally standardised data collection instruments; and
- diverse strategies to assess pre-disease household income as the denominator for assessing whether costs due to TB were catastrophic.

As costs due to TB increase, the risk of adverse TB treatment outcomes (principally treatment non-completion) increases. Indeed, we found that in Peruvian shantytowns when costs due to TB exceeded 20% of the pre-illness income of that household, then treatment outcomes were more likely to be adverse (treatment non-completion, treatment failure or death during treatment) than favourable (cure or treatment completion) ^3^. Similar findings have been reported in Brazil and Moldova ^4, 5^. Consequently, costs due to TB are usually considered to be catastrophic if they exceed a threshold of 20% of the pre-illness household annual income ^6^, although other thresholds have been used occasionally ^7, 8^.

In 2011 the World Health Organisation (WHO) together with the Japan Anti-Tuberculosis Association (JATA) developed an international tool to estimate costs due to TB over a recent brief period (e.g. one month) and then extrapolate these costs to the entire TB disease and treatment duration. This venture led to the creation of a standardised handbook using this approach for conducting TB patient cost surveys that has been used in several countries ^2^.

The WHO “End TB Strategy” has three principal targets to be achieved by 2035: a 95% reduction in the number of TB deaths; a 90% reduction in TB incidence rate; and 0% of TB-affected families facing catastrophic costs due to TB ^9^. The United Nations “Sustainable Development Goals” (SDG) describe similar targets to be achieved by 2025: a 90% reduction in the number of TB deaths; an 80% reduction in TB incidence rate; and 0% of TB-affected families facing catastrophic costs due to TB ^9^. Thus, both WHO and SDG global objectives prioritise eliminating catastrophic costs due to TB. This is generally believed to require sufficient political action that TB-affected patients and their TB-affected households can:

- reduce direct costs of out-of-pocket expenditures due to TB;
- reduce indirect costs by maintaining their income as much as possible despite TB; and also where necessary
- receive socioeconomic support to reduce the impact of costs due to TB.

Preventing catastrophic costs due to TB has been prioritised in global policy in order to:

- mitigate the adverse effects of TB on quality of life ^10^;
- reduce the impoverishing effects of TB ^1^; and
- increase the likelihood that patients with TB will be able to afford to complete TB care sufficiently to be permanently cured and return to good health ^11^.

Despite the consensus that catastrophic costs due to TB should be prevented, there is remarkably little clarity concerning how this may best be achieved. For example, from first principles it seems logical that interventions including the following may reduce catastrophic costs due to TB.

### Earlier TB case-detection

- Improved health systems and active case finding searching for people with TB disease (instead of passive case finding, waiting for them to present to and be diagnosed by health facilities) may more often diagnose TB earlier in the disease, whilst it is less severe and has caused less costs.

### Reducing TB severity

- Reductions in TB drug-resistance and co-morbidities and optimisation of therapy can reduce the costs due to TB and therefore reduce the incidence of catastrophic costs due to TB.

### Reduced costs during TB therapy

- Education, public health promotion, stigma reduction, laws and other measures may further reduce the indirect costs of lost employment due to TB.
- Information, improved health systems and universal health coverage may help to reduce the direct out-of-pocket expenditures caused by TB disease.
- Providing home-based care versus community-based clinic care versus hospital-based care in order to potentially reduce direct and indirect costs due to TB.

### Supporting TB-affected households

- TB specific socioeconomic support for people with TB disease may mitigate and/or reimburse their direct and indirect costs due to TB.
- Existing socioeconomic support systems (such as microcredit or cash transfer interventions to reduce extreme poverty) may be sensitive to, or be made sensitive to people living with TB, for example by adding TB disease to their eligibility criteria.

### Increasing pre-TB income

- Socioeconomic development may decrease poverty sufficiently to reduce the risk that costs due to TB reach the threshold for catastrophic costs.

### Reducing TB cases

- Reductions in poverty, under-nutrition, HIV, and other factors together possibly with improved public health systems may reduce the incidence of TB and hence indirectly reduce the incidence of catastrophic costs due to TB.

We have modelled the potential global effects of TB-specific versus TB-sensitive interventions ^12^ and have in Peru been prospectively evaluating the health and economic effects of TB-specific socioeconomic interventions for TB-affected households ^13– 17^. Related findings have been reported in other settings ^18^. Ecological analyses ^19^ and modelling ^20^ studies have further assessed the impact of social protection interventions on TB. For the current research, in order to inform public health policy, we aim to complete a systematic review and meta-analysis of these and other approaches to eliminate catastrophic costs due to TB.

### Objectives

The objectives of this study are to do a systematic review and meta-analysis of interventions aiming to eliminate catastrophic costs due to TB.

### Review questions

The questions of this systematic review and meta-analysis are what:

- strategies have been used to eliminate catastrophic costs due to TB; and
- is the effectiveness of these interventions for eliminating catastrophic costs due to TB?

## Methods

This systematic review and meta-analysis will follow the Preferred Reporting Items for Systematic Reviews and Meta-Analyses (PRISMA-P) checklist. The protocol is registered in the PROSPERO database 2022 CRD42022292410 available from: https://www.crd.york.ac.uk/. The individual link for this record is: https://www.crd.york.ac.uk/prospero/display_record.php?ID=CRD42022292410).

The eligibility criteria for studies to be included in this study are as follows.

### Inclusion criteria

Studies concerning the elimination of catastrophic household costs due to TB, including any type of TB (pulmonary or extrapulmonary; drug-susceptible or drug-resistant; whether or not complicated by comorbidities such as associated HIV-infection).

### Exclusion criteria

Studies that could not inform strategies to achieve the WHO and United Nations SDG target of eliminating catastrophic costs due to TB because the study only quantified:

- out-of-pocket expenditure costs without considering indirect costs of lost income; or
- monetary costs without assessing these costs as a proportion of household income; or
- catastrophic costs at a population level without considering the proportion of individual households that experienced catastrophic costs.

### Population

The population to be included in this systematic review and meta-analysis is TB-affected households i.e. patients with TB and the people living with them.

### Intervention/Exposure

Interventions will include any strategies aiming to mitigate or eliminate catastrophic costs due to TB e.g. TB active case finding (versus standard of care passive case finding); socio-economic support (compared with standard of care without socio-economic support); or home-based care (compared with standard of care in hospital).

### Comparison

The comparison / control condition will be standard of care (without any intervention).

### Outcome

The proportion of households with catastrophic costs due to TB.

### Information sources

Three electronic databases will be searched: PubMed, Scopus and Web of Science. We will also search reference lists from relevant publications.

### Search strategy

We will use the following search terms:

Pubmed: ((tuberculosis[MeSH Terms]) OR (tuberculosis OR koch disease* OR TB[Title/Abstract]))

AND (catastrophic cost* OR catastrophic household cost*[Title/Abstract])

Scopus: TITLE-ABS-KEY ((tuberculosis OR "koch disease" OR tb) AND (catastrophic AND cost*))

Web of science: (tuberculosis OR Koch disease* OR TB) AND (catastrophic AND cost*) (All Fields)

### Measures of effect

The main measure of effect will be the proportion of households with catastrophic costs due to TB. For continuous or categorical data outcomes, we will use mean or rate differences between the catastrophic cost intervention group versus the control group. For dichotomous data outcomes, odds ratio, relative risk, and/or absolute risk will be used. For data measured on the same scale and the same unit, weighted mean differences will be used, otherwise standardised mean differences will be used. The 95% confidence intervals of these measures will also be assessed.

### Data extraction

Data will be extracted from studies selected from the electronic databases using the search strategy. We will also review the references cited by these publications to find other relevant articles. Two reviewers will independently review potentially relevant publication titles, then abstracts and finally full-text publications for eligibility. Discrepancies will be resolved by discussion and when necessary independent consideration by another reviewer. The following data will be extracted from each publication:

- the proportion of households with catastrophic costs due to TB;
- the proportion of households with frequently-occurring study characteristics e.g. sociodemographic factors, TB diagnostic test used, TB treatment administered first-line versus second-line, type of TB pulmonary versus extra-pulmonary;
- the proportion of studies using frequently-reported strategies for quantifying out-of-pocket direct costs e.g. a one-time questionnaire versus a monthly questionnaire versus a questionnaire applied three times (at the beginning of treatment, the end of intensive phase and the end of treatment);
- the proportion of studies using frequently-reported strategies for quantifying indirect cost of lost income e.g. self-report versus calculation using the human capital approach;
- the proportion of studies using frequently-reported strategies for quantifying pre-illness household income e.g. self-report versus using the World Bank poverty headcount;
- the proportion of studies reporting strategies for quantifying whole illness costs and their timing e.g. prospectively by repeated questionnaires versus extrapolation from a one-month period using the WHO approach;
- the proportion of studies reporting variables known to be related to catastrophic costs (e.g. more versus less poor);
- the proportion of studies reporting each type of intervention aiming to eliminate catastrophic costs e.g. active case finding versus passive case finding; economic and social support versus standard of care;
- the proportion of studies reporting each methodology used to assess the impact of interventions aiming to eliminate catastrophic costs e.g. randomised controlled trial versus observational studies;
- the magnitude of impact and statistical significance of interventions aiming to eliminate catastrophic costs due to TB.

The data will be extracted in CSV format that will be uploaded to the Rayyan software to screen for duplicate documents as well in a Microsoft Excel spreadsheet document. The study selection process will be documented using the PRISMA flow diagram. Heterogeneity of data will be assessed if there are enough suitable data to perform a meta-analysis. A shared cloud-based spreadsheet will log all edits and who makes them.

### Type of studies

We will include all types of studies that inform the review objectives, without any restriction. For example: observational quantitative, qualitative, and mixed methods studies; intervention studies including randomised controlled trials; reviews; editorials; perspectives; and mathematical modelling studies will be extracted.

### Risk of bias (quality) assessment

We anticipate that the quality assessment tool for case control studies from the National Heart, Lung, and Blood Institute (NHLBI) may be most appropriate to generate an overall rating for the quality of each study of “good”, “fair”, or “poor”. This tool is available from the “Quality Assessment of Case-Control Studies” tool at the following link:

https://www.nhlbi.nih.gov/health-topics/study-quality-assessment-tools).

Depending on pilot work after the data have been extracted, an alternative tool may be used such as Version 2 of the Cochrane risk-of-bias tool for randomised trials. These plans may be modified if necessary, as adaptations to the progress of the systematic review.

### Strategy for data synthesis

As defined above, the principal measure of effect for this study will be to compare the proportion of households with catastrophic costs due to TB for intervention versus control groups. All proportions will be presented as percentages. Firstly, the raw data actual proportion of households with catastrophic costs due to TB will be compared in graphical and/or tabular form for both the intervention and also for the control group in each study. Secondly, the analysed data i.e. the odds ratios (and/or relative risks) of households experiencing catastrophic costs due to TB in intervention versus control groups will be compared in graphical and/or tabular form for each study. Proportions (and whenever possible odds ratios and/or relative risks) will be presented with their 95% confidence intervals. Whenever possible, comparisons will be reported as statistically significant (P<0.05) or not. 

### Meta-analysis

If we find sufficiently similar and suitable intervention studies, then we will assess the heterogeneity of the data with I ^2^ statistics and a Forest plot graph. All data will be analysed using Stata Software version 16.0 (Stata Corporation LLC, College Station, Texas, USA). The meta-analyses will include pooled odds ratios of comparable studies calculating the respective weighted means of these ratios, including weighted confidence intervals.

## Ethics and dissemination

Approval from an Ethics committee will not be required for this systematic review and meta-analysis because it will include analysis of only anonymous unlinked data. We intend to present this work at conferences and to publish it in an international peer-reviewed open-access journal.

## Discussion

The COVID-19 pandemic is believed to be markedly increasing TB disease, adverse TB outcomes, catastrophic costs due to TB and poverty ^21^, whilst impairing TB case finding and cure. We hope that this systematic review and meta-analysis will help to inform strategies for reducing or potentially eliminating catastrophic costs due to TB, towards ending TB.

## Data Availability

No data are associated with this article. The PRISMA-P checklist for “A protocol for a systematic review and meta-analysis of strategies to quantify or eliminate catastrophic costs due to tuberculosis” is available from the Harvard Dataverse: https://doi.org/10.7910/DVN/JS3GVY/DKK8LN21 It is also available with a CC BY 4.0 licence from the IFHAD: Innovation For Health And Development data repository: http://www.ifhad.org/wp-content/uploads/2021/12/Catastrophic_costs_search_strategy.pdf Data are available under the terms of the Creative Commons Zero "No rights reserved" data waiver (CC0 1.0 Public domain dedication).
