# Supplementary material for: Interventions aiming to eliminate catastrophic costs due to tuberculosis: a protocol for a systematic review and meta-analysis
Source: Wellcome Open Res. 2022 Nov 3;7:92. Originally published 2022 Mar 15. [Version 2] doi: 10.12688/wellcomeopenres.17521.2 (PMC10170179; doi:10.12688/wellcomeopenres.17521.2)
Supplement: Peer review history

# Peer review: Reviewer response for version 2

**DOI:** 10.21956/wellcomeopenres.20392.r56506

**Published:** 2023-06-06

**Authors:** Shweta R. Singh

**Affiliations:** 1 Saw Swee Hock School of Public Health, National University of Singapore, Singapore, Singapore

Overall, a meaningful pursuit of finding interventions aimed at eliminating catastrophic costs. A few methodological clarifications and consistency (in the Prospero protocol and this manuscript) can help the systematic review. 

 A few of the concerns are raised below:

1. The paper has many bullet points, which I am not sure are conventional in scientific papers. It would perhaps be better to put these together in a paragraph format. 
2. The protocol registered in the PROSPERO database seems not updated compared to this version. 
3. There is no plan for data synthesis from qualitative and mixed-methods articles. 
4. Also, the data extraction parameters from reviews, editorials, and mathematical modelling studies are not stated. 
5. For Risk of bias (quality) assessment, do you mean you will use separate tools for observational (cross-sectional, case-control and cohort) studies and controlled trials? 
6. The PRISMA-P checklist link is not working in the data availability section. 

Is the study design appropriate for the research question?

Yes

Is the rationale for, and objectives of, the study clearly described?

Yes

Are sufficient details of the methods provided to allow replication by others?

Partly

Are the datasets clearly presented in a useable and accessible format?

Not applicable

Reviewer Expertise:

Infectious diseases, Antimicrobial resistance, epidemiology

I confirm that I have read this submission and believe that I have an appropriate level of expertise to confirm that it is of an acceptable scientific standard.

# Peer review: Reviewer response for version 2

**DOI:** 10.21956/wellcomeopenres.20392.r56279

**Published:** 2023-05-17

**Authors:** Bianca Sossen

**Affiliations:** 1 Department of Medicine, Faculty of Health Sciences, University of Cape Town, Rondebosch, Western Cape, South Africa

Carballo-Jimenez and colleagues have presented a study protocol for a systematic review that will summarise all published interventions that aim to eliminate catastrophic costs due to tuberculosis, and the effectiveness of these interventions. The protocol is clearly written and plans to follow PRISMA guidelines. 

 I have the following minor recommendations for consideration:

1. As part of data extraction, when the authors say "first-line versus second-line" (TB treatment), do they mean drug-susceptible versus drug-resistant?
2. In light of the fact that the protocol only plans to review published articles, suggest including a planned review for any publication bias with a funnel plot as part of your data synthesis strategy
3. The link to the PRISMA-P checklist does not work

Is the study design appropriate for the research question?

Yes

Is the rationale for, and objectives of, the study clearly described?

Yes

Are sufficient details of the methods provided to allow replication by others?

Yes

Are the datasets clearly presented in a useable and accessible format?

Not applicable

Reviewer Expertise:

Tuberculosis diagnostics and natural history

I confirm that I have read this submission and believe that I have an appropriate level of expertise to confirm that it is of an acceptable scientific standard.

# Peer review: Reviewer response for version 2

**DOI:** 10.21956/wellcomeopenres.20392.r56508

**Published:** 2023-05-09

**Authors:** Jane Rahedi Ong'ang'o

**Affiliations:** 1 Centre for Respiratory Disease Research, Kenya Medical Research Institute, Nairobi, Kenya

Thanks for the opportunity allowing me to review this protocol. The title is clear and it indicates that this is a protocol for systematic review. There is a clear definition of what type of studies will be included in the review based on the catastrophic experience and whether this exceeds a threshold of 20% of the pre-illness household annual income as recommended by WHO. Various strategies (interventions) to eliminate catastrophic expenditure have been described in the introduction as well as the control group and the primary study outcome. Search strategies and data management is described clearly. Measures for taking care of risk for bias for individual studies has been stated. Well written protocol.

Is the study design appropriate for the research question?

Yes

Is the rationale for, and objectives of, the study clearly described?

Yes

Are sufficient details of the methods provided to allow replication by others?

Yes

Are the datasets clearly presented in a useable and accessible format?

Not applicable

Reviewer Expertise:

Public Health/Epidemiology Research in tuberculosis/HIV with a focus in supporting health system strengthening

I confirm that I have read this submission and believe that I have an appropriate level of expertise to confirm that it is of an acceptable scientific standard.

# Peer review: Reviewer response for version 2

**DOI:** 10.21956/wellcomeopenres.20392.r53176

**Published:** 2022-12-01

**Authors:** Sedona Sweeney

**Affiliations:** 1 Department of Global Health and Development, London School of Hygiene & Tropical Medicine, London, UK

I have had a look and the paper looks much improved, I have no further comments. I look forward to reading the results of this very interesting literature review!

Is the study design appropriate for the research question?

Yes

Is the rationale for, and objectives of, the study clearly described?

Partly

Are sufficient details of the methods provided to allow replication by others?

Partly

Are the datasets clearly presented in a useable and accessible format?

Not applicable

Reviewer Expertise:

health economics with a focus on tuberculosis in low-income and middle-income settings

I confirm that I have read this submission and believe that I have an appropriate level of expertise to confirm that it is of an acceptable scientific standard.

# Peer review: Reviewer response for version 1

**DOI:** 10.21956/wellcomeopenres.19373.r50128

**Published:** 2022-08-04

**Authors:** Sedona Sweeney

**Affiliations:** 1 Department of Global Health and Development, London School of Hygiene & Tropical Medicine, London, UK

Thank you for the opportunity to review this protocol for a systematic review on catastrophic costs due to TB. This is an important topic for which there is still very little existing evidence.

- I would recommend clarifying the research question as described in the study. The objective of the study is described as ‘to assess the evidence for strategies to quantify or eliminate catastrophic costs due to TB’. Given this description, it is unclear to me whether the primary focus of the review will be on quantifying the prevalence of catastrophic costs, or on the impact of interventions aiming to reduce or eliminate catastrophic costs (such as active case finding, home-based care, or socioeconomic support).
- If the main aim is to quantify the prevalence of catastrophic costs, this may not be a substantial addition to the literature as this question has recently been addressed by other authors (see Ghazy, R.M *et al.*, 2022) ^1^.
- I would therefore recommend considering focusing this paper on the impact of interventions aiming to reduce or eliminate catastrophic costs, an important and interesting question for which there is very little existing evidence. I would recommend including some further background describing any existing evidence on this topic in the introduction, to justify the rationale for the study.
- This study is planned as a systematic review and meta-analysis. The search strategy for this review is well-described. The description of plans for data extraction could be improved with further detail, for example by describing which study and methodological characteristics will be extracted, and which variables known to be related to catastrophic costs will be considered.
- The description of the risk of bias is clear, however the link to the NHLBI study quality assessment tool is broken and needs amending. The description of methods for data synthesis should describe synthesis methods for all measures of effect included in the study.

Is the study design appropriate for the research question?

Yes

Is the rationale for, and objectives of, the study clearly described?

Partly

Are sufficient details of the methods provided to allow replication by others?

Partly

Are the datasets clearly presented in a useable and accessible format?

Not applicable

Reviewer Expertise:

health economics with a focus on tuberculosis in low-income and middle-income settings

I confirm that I have read this submission and believe that I have an appropriate level of expertise to confirm that it is of an acceptable scientific standard, however I have significant reservations, as outlined above.

## References

[1] : A systematic review and meta-analysis of the catastrophic costs incurred by tuberculosis patients. *Scientific Reports* .2022;12(1) : 10.1038/s41598-021-04345-x 10.1038/s41598-021-04345-x 35017604PMC8752613

# Response

**Published:** 2022-09-10

**Authors:** Carlton Evans

**Affiliations:** IFHAD: Innovation For Health And Development, Peru

Thank you for these helpful suggestions. 

 Precisely as the reviewer suggested, we have clarified the wording of the title, focus and research question of the protocol to increase their consistency with the unchanged wording of our primary objective. These changes make the absence of overlap with previous systematic reviews clear.

 As the reviewer suggested, we have also: included additional background information in the ‘Introduction’ section; our description of plans for data extraction and data synthesis have been improved; the risk of bias link has been clarified.

 We believe these improvements fully address the reviewer’s observations and will be pleased to make any other necessary changes.
